# Relationship between Problematic Internet Use and Depression in Young Latin American College Students

**DOI:** 10.3390/bs14080719

**Published:** 2024-08-16

**Authors:** Andrea Vázquez-Martínez, Verónica Villanueva-Silvestre, Beatriz Abad-Villaverde, Cristina Santos-González, Antonio Rial-Boubeta, Víctor José Villanueva-Blasco

**Affiliations:** 1 Faculty of Health Sciences, Valencian International University, 46002 Valencia, Spain; avazquezm@universidadviu.com (A.V.-M.); vvillanueva@universidadviu.com (V.V.-S.); cristina.santos.g@professor.universidadviu.com (C.S.-G.); 2 Research Group on Health and Psycho-Social Adjustment (GI-SAPS), Valencian International University, 46002 Valencia, Spain; 3 Faculty of Humanities and Education, Universidad Nacional Pedro Henríquez Ureña, Santo Domingo 10203, Dominican Republic; babad@unphu.edu.do; 4 Faculty of Psychology, University of Santiago de Compostela, 15782 Santiago de Compostela, Spain

**Keywords:** depression, problematic Internet use, college students, Latin America

## Abstract

(1) Problematic Internet use (PIU) in young people is a topic of great interest both in the field of addictions and mental health, but scientific evidence is limited in Latin America. The aim was to analyze the relationship between PIU and depression in Latin American college students. (2) Methods: The sample consisted of 1828 college students (63.7% women), aged between 18–30 years (M = 21.64 years). (3) Results: PIU was detected in 40.2% of cases, and severe or moderately severe depression in 31.7%. Rates of severe depression in students with PIU were 3.02 times higher than in those without PIU (χ2(3) = 168.443; *p* < 0.000). The presence of PIU was also statistically significantly higher among youth with depressive symptoms. Linear and logistic regression models for predicting PIU, show how the depression level constitutes a risk factor for PIU: seven times higher for severe depression; more than five times higher for moderate depression; and more than two times for mild depression. (4) Conclusions: There is a clear association between depression and PIU, suggesting that a higher level of depression would act as a predictor of PIU. However, this finding is exploratory. Future studies should clarify the directionality of the relationship between both variables.

## 1. Introduction

The relationship between problematic Internet use (PIU) and psychological well-being and mental health has been widely debated in the last decade. There are studies that link PIU to emotional disorders such as depression, anxiety, and stress [1,2].

PIU is a topic of great interest for both institutions and researchers, but it remains particularly controversial, due to the lack of consensus on its naming [3,4,5]. PIU could be understood as a behavior that can cause problems related to Internet use, which turn into a dysfunctional pattern with severe effects on the well-being of people, especially young adults and adolescents [6].

College students are also an important vulnerable group [7]. Pal et al. [8] indicate wide access to the Internet through different devices available to college students and the fact that they enjoy greater freedom, both of which enable excessive and maladaptive use, as reasons for a higher risk of PIU. Although studies on PIU in adolescents, young people, and college students are numerous worldwide, in Latin America, the studies are proportionally scarce and generally have small sample sizes.

However, in Latin America, empirical studies on problematic Internet use (PIU) do not indicate prevalence rates as high as those found in the studies from other countries [9,10]. Different studies of the young population have indicated PIU rates of up to 7% in Colombia and Uruguay [11] and close to 3% in Brazil [12]. In any case, beyond the possible geographical and cultural differences, various authors warn of the confusion on this issue, stemming from the persistent lack of consensus, both in conceptual and methodological terms, which leads to a dangerous tendency to overpathologize the problem [13,14,15].

The study by Villanueva-Blasco et al. [16] analyzed the frequency of Internet usage and the Internet usage habits in populations from Ecuador, Peru, Colombia, and Argentina. Among young people aged 18 to 29, between 84% and 90.2% showed an Internet connection every day or almost every day, and around 52% connected for 5 h or more per day. These patterns of high Internet connectivity and usage hours have been associated with the development of PIU. However, this is not the only health risk stemming from this pattern of Internet use. Lam and Peng [17] indicate that people with PIU are at a 2.5 times higher risk of suffering some depression disorder. Monezi et al. [18], in a study of young Brazilian college students, found that 4.8% of participants were cataloged as high-risk Internet users and that their risk of severe depression symptoms was 10 times higher than that of the group cataloged as Internet users with no risk. Belchior et al. [19], in a study of the Brazilian university population, indicate a higher prevalence of PIU among students with some kind of mental disorder, such as depression, than that among those who did not have this problem.

The direction of this relationship is a focus of research interest, given the repercussion it can have in the understanding of the dynamics between both problems. Some studies support the idea that Internet use, or more specifically, Internet and social networks use, produce certain negative emotions [20]. Ciarrochi et al. [21] found that PIU predicted poor mental health development, while poor mental health did not predict PIU development. Despite this, experts recommend maintaining a more cautious attitude when drawing conclusions regarding the relationship between PIU and psychological well-being, since it is also possible to defend the hypothesis of a relationship in the opposite direction: emotional distress may be a good predictor or antecedent of a problematic or maladaptive use of technology [22,23]. 

Studies that analyze the relationship between PIU and depression are scarce within the Latin American population and do not delve into aspects such as the level of depression or sociodemographic aspects, such as sex or age. The purpose of this study was (1) estimating the PIU rate among Latin American college students, focusing on sex and age, and (2) analyzing the PIU’s relationship with depression.

## 2. Materials and Methods

### 2.1. Participants

The initial sample consisted of 1997 college students. Of them, 169 (8.5%) were removed because of missing values, incoherent response patterns, or being outside the established age range (18–30 years old). The final sample consisted of 1828 college students (63.7% women and 36.3% men) aged between 18 and 30 years (M = 21.64; SD = 3.2) and coming from four different countries: Dominican Republic, Ecuador, Mexico, and Peru.

### 2.2. Instruments

The sociodemographic variables considered were (a) sex (men, women); (b) age (18–21 years, 22–25 years, and 26–30 years); (c) country.

Depression was evaluated with the PHQ-9 (Patient Health Questionnaire) [24,25], Spanish adaptation [26]. The scores were added together, and a total score between 0 and 27 is obtained. The cut-off points [27] are 0–4: minimal depression, 5–9: mild depression, 10–14: moderate depression, 15–19: moderately severe depression, and 20–27: severe depression. In this study, α PHQ-9 = 0.84.

Problematic Internet use (PIU) was evaluated with the EUPI-a [28]. The scores are added up, and a total score between 0 and 44 is obtained. The cut-off point for setting PIU is 16. In this study, Cronbach’s α = 0.85 and McDonald’s omega coefficient = 0.86.

### 2.3. Procedure

The data collection strategy was based on a survey hosted on a website, posts on social media, and advertisements via e-mail and smartphone messaging applications. The participants were informed that involvement was voluntary and asked to give their consent to participate.

### 2.4. Data Analysis

Data analysis was performed with IBM SPSS Statistics for Windows, version 25 (IBM Corp., Armonk, NY, USA), for all analyses except to calculate McDonald’s omega, which was carried out with The Jamovi Project [29] for Windows version 2.3.9. As a first step, the assumptions of normality (Kolmogorov–Smirnov) and homoscedasticity (Levene) were checked. Student’s *t*-tests, considering sex as an independent variable, were applied to compare PIU and depression means, and analyses of variance (ANOVAs) were conducted to compare age group differences. Contrasts with the Bonferroni correction to assess the differences between pairs were carried out retrospectively. In addition, frequency analysis and the chi-squared test were also performed for intragroup differences (disaggregated by sex and age) in the prevalence of PIU and PHQ-9. For the comparison of means on the PHQ-9 between the groups with and without PIU, Student’s *t*-test was applied, after checking compliance with the assumptions of normality (Kolmogorov–Smirnov) and homoscedasticity (Levene). A correlation analysis was also carried out (Pearson’s bivariate correlation for metric variables and Spearman’s order correlation for ordinal variables). The phi (φ) coefficients and the contingency coefficients (CCs) were performed to estimate the effect size and Goodman and Kruskal’s tau (τ) to analyze the directional effects of the relationship between PIU and depression. Based on the latter, a simple linear regression analysis was performed with depression as an independent variable and PIU as a criterion variable. Finally, univariate and multivariate logistic regression analyses, adjusted for sex, age, and level of depression, were performed to predict PIU.

## 3. Results

Table 1 shows that 40.2% (*n* = 735) had PIU. According to sex, no statistically significant differences were found (χ2(1) = 0.306; *p* = 0.580), also not according to age (χ2(2) = 5.899; *p* = 0.052), although the highest percentages again correspond to the 18–21 age group.

Regarding the presence of depression in the sample of college students (Table 2), 41% of surveyed students had mild depression, 28% had moderate depression, and 3.7% had severe depression. Per sex, depression prevalence was higher among women than among men (χ2(3) = 52.227; *p* < 0.000). According to age, the 18–21 age group had the highest prevalence of depression, followed by the 22–25 age group, and, finally, the 26–30 age group (χ2(6) = 20.495; *p* < 0.002). 

Similar results according to sex and age can be found if we compare the overall scores on the PIU and PHQ-9 scales (Table 3 and Table 4), with the only exception that, in this case, it is possible to establish significant differences at the PIU level between the three age groups (F(1827) = 8.37; *p* < 0.001). In the comparison of depression, statistically significant differences were found between men and women (t(1826) = 6.21; *p* < 0.001) and between age groups (F(1827) = 9.50, *p* < 0.001). Post hoc analysis showed that differences for PIU were found between the age ranges of 18–21 y/o and 26–30 y/o (*p* < 0.001), while, for depression, they were observed between the groups of 18–21 y/o and 26–30 y/o (*p* < 0.001) and between the groups of 22–25 y/o and 26–30 y/o (*p* < 0.001).

In regard to the association between PIU and depression, a positive correlation can be observed between both variables (r = 0.37; *p* < 0.01), resulting in a statistically significant higher mean PHQ-9 score (t(1412.837) = −13.682; *p* < 0.000); d = 0.66) among college students with PIU (M = 9.95, SD = 5.425) compared to those who had no PIU (M = 6.59, SD = 4.674). As can be seen in Table 5, depression levels were statistically significantly higher among students with PIU (χ2(3) = 168.44; *p* < 0.000), with the percentage of cases with severe depression almost three times higher (6% vs. 2.2%).

**Figure 1:**
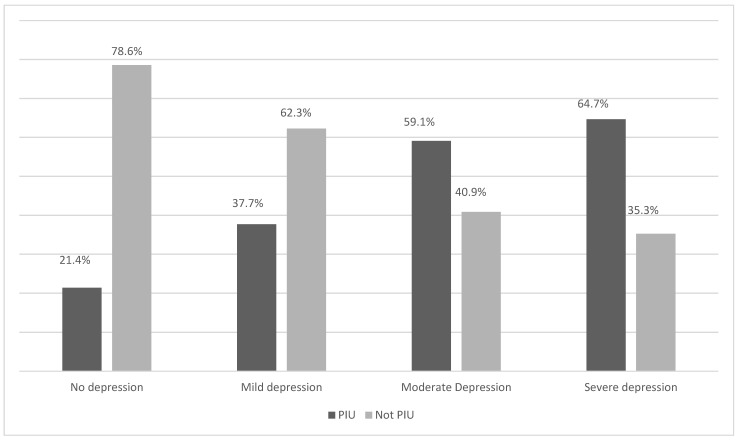
Prevalence of PIU depending on the level of depression.

If both axes are exchanged in the analysis, a progressive increase can be observed for the PIU rate according to the depression level indicated by the PHQ-9 (Figure 1). The PIU rates increase from 21.4% for non-depression to 64.7% for severe depression.

Regarding the directionality of this relationship, the Goodman and Kruskal tau value is 0.092 when PIU is considered as a dependent variable and 0.035 when it is depression. Based on this result, a simple linear regression analysis was carried out, which found that depression was a significant predictor of PIU (β = 0.369; t1826 = 16.99; *p* < 0.001), with a coefficient of determination of 0.137. A logistic regression analysis was carried out too, from both a univariate and a multivariate perspective (Table 6). As result, the sex variable was not relevant, either in the univariate or in the multivariate analysis. Regarding age, the group of 26–30 y/o presented a lower risk rate [0.72 (95% CI: 0.54–0.96)] of developing PIU than the reference group (18–21 y/o) according to the univariate analysis, although, later, this relevance disappeared in the multivariate analysis. Third, it was observed that the higher the level of depression, the higher the risk of PIU. Taking college students without depression as the reference, according to the univariate analysis, those college students with mild depression showed a 2.21 times higher rate of developing PIU (95% CI: 1.71–2.88), which was slightly higher in the multivariate analysis, with a rate 2.26 times higher (95% CI: 1.73–2.93). On the other hand, students with moderate depression showed a 5.31 times higher rate of developing PIU (95% CI: 4.02–7.00), which increased in the multivariate analysis to a rate 5.42 times higher (95% CI: 4.09–7.19). Finally, students with severe depression showed a 6.73 times higher rate of developing PIU (95% CI: 3.92–11.57), which increased in the multivariate analysis to a rate 6.93 times higher (95% CI: 4.02–11.94).

## 4. Discussion

The purpose of this study was to estimate the PIU rate among Latin American college students and to analyze the PIU’s relationship with depression. 

The first highlight is that 40.2% of the overall sample could have PIU. Women (40.6%) had a very similar rate compared to men (39.4%). Considering age, the differences found for PIU, although not reaching statistical significance, were consistent with those of Vega et al. [30] or Villanueva-Silvestre et al. [2], who obtained higher rates for the younger group.

In recent years, there have been warnings on the increase in PIU rates worldwide. However, different authors warn of the danger of overpathologizing this type of behavior [13]. Nogueira-López et al. [15] point out the variability in prevalence rates depending on whether scales based on the DSM-5 [31] or ICD-11 [32] substance use criteria are used, with the latter being more realistic and reliable. However, one of the limitations of the present study is the sample imbalance between participating countries and that it is a convenience sample and, therefore, is not representative. The prevalence of PIU found should be considered on an exploratory basis. Having data from Latin America can contribute to a better conceptualization as a measurement of the PIU.

As for the depression rate among Latin American college students, it was 28% for moderate depression and 3.7% for severe depression. These results are in line with previous research works that have depression rates among the Latin American university population [33]. Taking into account sex, regardless of the level of depression, women had a statistically significant higher mean score than men, which is in accordance with the previous research [2,34]. Regarding age, statistically significant differences were found for depression, with the score being higher inversely to the age range and the highest being the one for 18–21 age group. These data coincide with those found in a Spanish sample by Villanueva-Silvestre et al. [2]. Therefore, given the generality that it is the youngest who have higher levels of depression, it is worth asking whether we are facing a global mental health problem, and what are the reasons why it affects the youngest in a special way. Future studies should address this issue. 

In this study, the relationship between PIU and depression was confirmed again, with the average score of the PHQ-9 statistically significantly higher among students with PIU than among those who had no PIU. This finding reinforces the evidence presented by other studies [1,2]. The severe depression rate among college students was 2.7 times higher in case of PIU (6% vs. 2.2%), but more interesting may be the fact that the rate of PIU was 3.02 times higher in the case of a student with severe depression compared to that in one without depression. This prompts a deep reflection on the possible directionality of the relationship between PIU and depression. The Goodman and Kruskal tau values found lead us to think that PIU could be a consequence of emotional problems rather than an antecedent of them. This finding is confirmed by results from both linear and logistic regression models for predicting PIU, which clearly show how the depression level constitutes a risk factor for PIU, being almost seven times higher for severe depression, more than five times higher for moderate depression, and more than two times for mild depression. However, the cross-sectional design of the present study does not allow it to be confirmed with complete certainty. 

The results should be generalized with caution as there are some limitations associated with possible coverage errors and randomness of the sample, due to the use of an online survey, even though actions were taken to compensate for these errors. Likewise, although our sample was large, it cannot be considered representative of the Latin American population. The findings of the present study should be considered in exploratory terms; they are not epidemiological or confirmatory of the suggested relationship between depression and PIU.

Beyond the mentioned limitations, this type of empirical research is increasingly necessary, especially in Latin America, a region in which this field of study has been less explored compared to Asia or Europe. The high prevalence of PIU found, not exclusively limited to children and adolescents, combined with its correlations with mental health, general health, and quality of life, makes PIU a public health issue that needs to be studied to offer effective responses. Empirically confirming in the present study with a sample of young Latin American students, the close relationship between PIU and a concerning issue such as depression, reaffirms the relevance of addressing this problem. However, the results obtained highlight some important challenges still to be resolved: the need to establish clear criteria for the diagnosis and screening of the problem, the analysis of intercultural, age, and gender differences, as well as the directionality of the relationship between PIU and health indicators, especially emotional health.

## 5. Conclusions

The findings of this study confirm the relationship between PIU and depression in Latin American university students. The main finding was that a higher presence of depression is associated with a greater risk of PIU, although this finding must be interpreted with caution due to the study’s limitations. Additionally, being male and between 18 and 21 years old increases the risk of PIU. There were no significant differences in PIU according to sex, but there were for depression, which was higher among women than among men. Age differences were observed for PIU, showing an inverse relationship, with younger individuals having higher average scores. In short, these findings contribute to the joint study of PIU and depression, providing data on Latin America, where studies are limited, and supporting the evidence found worldwide on the relationship between these two issues.

The findings of the present paper suggest several issues for discussion. Given the high prevalence of PIU observed in the present study, it is worth asking whether PIU should be considered in clinical terms. However, for a behavior to be considered an addiction, it must meet a number of requirements, such as clinical relevance, consistency of phenomenology with other addictive disorders, theoretical embeddedness, or taxonomic plausibility. But this shows the relevance of the subject and the care and rigor with which these issues should be treated. Furthermore, as indicated by some authors, there is a risk of overpathologization. Therefore, it is necessary to continue moving toward a consensus in the conceptualization of problematic uses of the Internet and how they are evaluated.

The analysis of the direction of the relationship between PIU and emotional health remains another unresolved question. The findings of the present study are in line with those that point to depression as a predictor of PIU [22,23]. However, in other studies [21], evidence in the opposite direction could be found (although to a lesser extent). Is a bidirectional relationship between both variables possible? Probably so.

## Figures and Tables

**Table 1:** Prevalence of PIU (entire sample, by sex and age).

	Global % (*n*)	Sex % (*n*)		χ^2^	Age % (*n*)			χ^2^
		Female	Male		18–21	22–25	26–30	
PIU	40.2 (735)	40.7 (474)	39.4 (261)	0.306	42.3 (454)	38.6 (191)	34.6 (90)	5.899

Note: PIU = problematic Internet use; χ^2^ = chi squared.

**Table 2:** Prevalence of depression (entire sample, by sex and age).

PHQ-9	Global % (*n*)	Sex % (*n*)		χ^2^ (CC)	Age % (*n*)			χ^2^ (CC)
		Women	Men		18–21	22–25	26–30	
No depression	27.4 (500)	21.9 (255)	37 (245)	52.227 *** (0.17)	24.6 (264)	28.7 (142)	36.2 (94)	20.495 ** (0.11)
Mild depression	41 (749)	42.6 (496)	38.2 (253)		41.8 (449)	39 (193)	41.2 (107)	
Moderate depression	28.0 (511)	31.4 (366)	21.9 (145)		30 (322)	27.9 (138)	19.6 (51)	
Severe depression	3.7 (68)	4.1 (48)	3 (20)		3.5 (38)	4.4 (22)	3.1 (8)	

Note: *** *p* < 0.001; ** *p* < 0.01; χ^2^ = chi squared; CC = contingency coefficient.

**Table 3:** PIU and PHQ-9 scores by sex.

	Women		Men		Comparison		Effect Size
	M	DT	M	DT	t	*p*	d
PIU	14.21	8.31	14.40	8.93	0.457	0.648	
PHQ-9	8.51	5.22	6.94	5.17	6.21 ***	<0.001	0.30 *

Note: *** *p* < 0.001; * *p* < 0.05; t = Student test; d = Cohen d.

**Table 4:** PIU and PHQ-9 scores by age.

	Age Groups	Descriptive		ANOVA					Post Hoc	
		M	DT	Sum of Squares		Df	MS	F	BONF	*p*
PIU	(1) 18–21	14.84	8.37	Between gr.	1210.10	2	605.05	8.37 ***	1 ≠ 3	<0.001
	(2) 22–25	14.02	8.91	Within gr.	131,891.81	1825	72.27			
	(3) 26–30	12.48	8.26	Total	133,101.91	1827				
PHQ-9	(1) 18–21	8.27	5.24	Between gr.	519.09	2	259.54	9.50 ***	1 ≠ 3 2 ≠ 3	<0.001 0.010
	(2) 22–25	7.88	5.32	Within gr.	49,875.88	1825	27.33			
	(3) 26–30	6.70	0.94	Total	50,394.97	1827				

Note: *** *p* < 0.001.

**Table 5:** Prevalence of depression depending on PIU.

	PIU % (*n*)	No PIU % (*n*)	χ^2^ (CC)
No depression	14.6 (107)	36 (393)	168.44 *** (0.29)
Mild depression	38.4 (282)	42.7 (467)	
Moderate depression	41.1 (302)	19.4 (209)	
Severe depression	6 (44)	2.2 (24)	

Note: *** *p* < 0.001; χ^2^ = chi squared CC = contingency coefficient.

**Table 6:** Logistic regression models to predict PIU.

PIU			
		Univariate POR (95% IC)	Multivariate ^1^ POR (95% IC)
Sex	Women	1	1
	Men	0.95 (0.78–1.15)	1.18 (0.96–1.45)
Age	18–21	1	1
	22–25	0.86 (0.69–1.07)	0.86 (0.69–1.09)
	26–30	0.72 (0.54–0.96)	0.84 (0.62–1.13)
Depression	No depression	1	1
	Mild depression	2.21 (1.71–2.88)	2.26 (1.73–2.93)
	Moderate depression	5.31 (4.02–7.00)	5.42 (4.09–7.19)
	Severe depression	6.73 (3.92–11.57)	6.93 (4.02–11.94)

Note: POR = prevalence of odds ratio; CI = confidence interval. ^1^ Adjusted for the other independent variables included in the column.

## Data Availability

The data that support the findings of this study are available from the corresponding author upon reasonable request.
